# Long-term evaluation of *Helicobacter pylori* screening in school health checkups: an 11-year study in Japan

**DOI:** 10.1007/s00535-025-02236-w

**Published:** 2025-03-07

**Authors:** Takuma Okamura, Akihiro Ito, Yugo Iwaya, Tadanobu Nagaya, Atsuhiro Hirayama, Hiroyoshi Ota, Taiji Akamatsu

**Affiliations:** 1 Department of Medicine, Division of Gastroenterology and Hepatology, Shinshu University School of Medicine, 3-1-1 Asahi, Matsumoto, Nagano 390-8621 Japan; 2 Department of Gastroenterology, Matsumoto Municipal Hospital, Matsumoto, Japan; 3 Endoscopic Examination Center, Shinshu University Hospital, Matsumoto, Japan; 4 Department of Biomedical Laboratory Sciences, School of Health Sciences, Shinshu University School of Medicine, Matsumoto, Japan; 5 Endoscopy Center, Nagano Prefectural Shinshu Medical Center, Suzaka, Japan

**Keywords:** High school, Adolescent, Eradication therapy, *Helicobacter pylori*, Gastric cancer

## Abstract

**Background:**

The eradication of *Helicobacter pylori* (*H. pylori*) at a younger age is considered effective in preventing gastric cancer. Toward this goal, we introduced primary *H. pylori* screening into routine high school health screenings in 2007. The present study aimed to elucidate the clinicopathological characteristics of *H. pylori*-infected students and evaluate the effectiveness of *H. pylori* screening in high school populations.

**Methods:**

Primary screening using a urinary anti-*H. pylori* antibody test was conducted on high school students from 2007 to 2017. Students who tested positive for this examination were recommended secondary screening by esophagogastroduodenoscopy (EGD), with eradication therapy for those with confirmed *H. pylori* infection. We analyzed data from 2007 to 2011 as the early period and from 2012 to 2017 as the late period.

**Results:**

Over 11 years, 5178 of 5193 (99.7%) subjects received primary screening, among which 184 students (3.6%) tested positive. The primary screening-positive rate decreased significantly from 4.7% in the early period to 2.8% in the late period (*p* < 0.01). EGD as secondary screening in 103 students (56%) revealed nodular gastritis (83.3%) as the most common endoscopic finding. *H. pylori* infection was diagnosed in 90 students (87.4%). The resistance rate of *H. pylori* to clarithromycin was 41.1%. The initial eradication therapy success rate by treatment selection according to *H. pylori* susceptibility was 96.5%.

**Conclusions:**

The introduction of *H. pylori* screening into school health checkups achieved high participation rates and appeared useful for identifying and treating *H. pylori* infection in young populations.

## Introduction

*Helicobacter pylori* (*H. pylori*) infection has been associated with future gastric carcinogenesis [1]. The eradication therapy for *H. pylori* has been shown to significantly reduce the incidence of gastric cancer [2, 3], with treatment at a younger age considered more effective in preventing cancer onset [4]. In recent years, the major route of *H. pylori* infection in Japan is thought to be intrafamilial transmission, especially between the mother and the child [5]. *H. pylori* eradication therapy at a young age is therefore important not only for the prevention of gastric cancer in patients themselves, but also for halting *H. pylori* spread to the next generation. As the rate of *H. pylori* infection in Japanese junior high school students is low at approximately 3% [6, 7], it is a challenge to efficiently identify afflicted individuals among adolescents. Furthermore, the precise usefulness and the safety of eradication therapy in teenagers remain uncertain.

The method of screening for *H. pylori* infection in young individuals and the most suitable age for treatment are controversial. Several trials on *H. pylori* screening and treatment without esophagogastroduodenoscopy (EGD) are underway in junior high schools across Japan [6–8] although long-term data are not yet available. We have been trailing the introduction of primary *H. pylori* screening with a urinary anti-*H. pylori* antibody test in routine school medical screenings for second-year high school students since 2007 [9]. The present study aimed to elucidate the clinicopathological characteristics of *H. pylori*-infected students and evaluate the usefulness of *H. pylori* infection screening in school health checkups through the results of over 10 years of testing.

## Methods

### Study design and subjects

Between 2007 and 2017, we conducted *H. pylori* screening as part of school health checkups. The subjects were second-year high school students (i.e., 16 or 17 years old) attending a single school in the city of Matsumoto, Nagano prefecture, Japan. Their body size was comparable to that of adults, which enabled eradication therapy at adult dosages through Japanese national insurance coverage. The primary screening method was a urinary anti-*H. pylori* antibody test (RAPIRAN®; Otsuka Pharmaceuticals, Tokyo, Japan), which was considered suitable for *H. pylori* screening even among adolescents due to its simplicity, low cost, non-invasiveness, and high sensitivity [10], as well as the fact that it leveraged urine samples already collected for routine school health checkups. In preparation for the primary screening, we developed an informational brochure that included basic information on *H. pylori*, the significance of early *H. pylori* detection, and details on this study including the right to refuse participation. The brochure was distributed to all participants and their guardians before screening to ensure comprehensive informed consent. Students who tested positive were requested to visit a medical facility by the school doctor. EGD was performed after obtaining informed consent from the student and parents at Shinshu University Hospital. We analyzed data from 2007 to 2011 as the early period and from 2012 to 2017 as the late period of this trial. This study was conducted according to the principles of the Declaration of Helsinki and was approved by the Ethics Committee of Shinshu University (no. 1997).

### Endoscopic findings

EGD was performed under sedation using 0.1 mg/kg of midazolam. Endoscopic findings were assessed by white-light endoscopy. The endoscopic degree of atrophic gastritis was classified according to Kimura–Takemoto classification [11].

### Assessment of *H. pylori* infection

*H. pylori* status was assessed by 5 biopsy specimens taken during EGD from each student: 1 each from the lesser (A1) and greater (A2) curvature of the antrum, 1 from the incisura angularis (IA), and 1 each from the lesser (B1) and greater (B2) curvature of the corpus according to the updated Sydney system (USS) [12]. The biopsy samples were stained with hematoxylin and eosin, and then immunostained for *H. pylori* with a rabbit anti-*H. pylori* polyclonal antibody (DAKO, Carpinteria, CA, USA) if necessary. The histological grades for neutrophil infiltration, mononuclear cell infiltration, atrophy, and intestinal metaplasia were judged as normal (0), mild (1), moderate (2), or marked (3) by an expert pathologist (HO).

One additional biopsy specimen each was taken from the greater curvature of the antrum and the gastric body to assess the antibiotic susceptibility of *H. pylori* isolates. The minimal inhibitory concentration breakpoints used were ≥ 1 μg/mL for clarithromycin (CAM) according to the value established by the Japanese Society of Chemotherapy [13] and ≥ 16 μg/mL for metronidazole (MNZ) based on the threshold determined by the European *H.* *pylori *Study Group [14]. All antimicrobial susceptibility tests for *H. pylori* were performed using the broth microdilution method.

*H. pylori* infection was deemed to be positive if either or both biopsies or cultures were positive, and absent if both tests were negative.

### Eradication therapy

The first-line eradication regimen was selected based on the results of antibiotic susceptibility. CAM-based eradication therapy, which consisted of a proton pump inhibitor (PPI) (rabeprazole 20 mg/day or esomeprazole 40 mg/day) or potassium-competitive acid blocker (P-CAB) (vonoprazan 40 mg/day), amoxicillin (AMPC) (1500 mg/day), and CAM (800 mg/day) for 7 days was given as the initial eradication therapy for patients carrying *H. pylori* strains susceptible to CAM. Otherwise, a MNZ-based regimen of PPI or P-CAB, AMPC, and MNZ (500 mg/day) for 7 days was selected for patients harboring CAM-resistant *H. pylori* strains, regardless of the strain’s susceptibility to MNZ. At 8 weeks after eradication therapy, treatment success was assessed by a ^13^C-urea breath test. Adverse events were recorded in interviews conducted by the attending physician either at the onset of symptoms or at the time of eradication therapy outcome assessment.

### Statistical analysis

Participation rate, *H. pylori* infection rate, and antibiotic resistance rate were evaluated using the χ^2^ test. Eradication rate was tested by per-protocol analysis. USS scores were expressed as mean values. All statistical analyses were performed using StatFlex software version 6.0 (Artech, Osaka, Japan). A p-value of < 0.05 was considered statistically significant.

## Results

### Participation rate and *H. pylori* infection rate

**Fig. 1:**
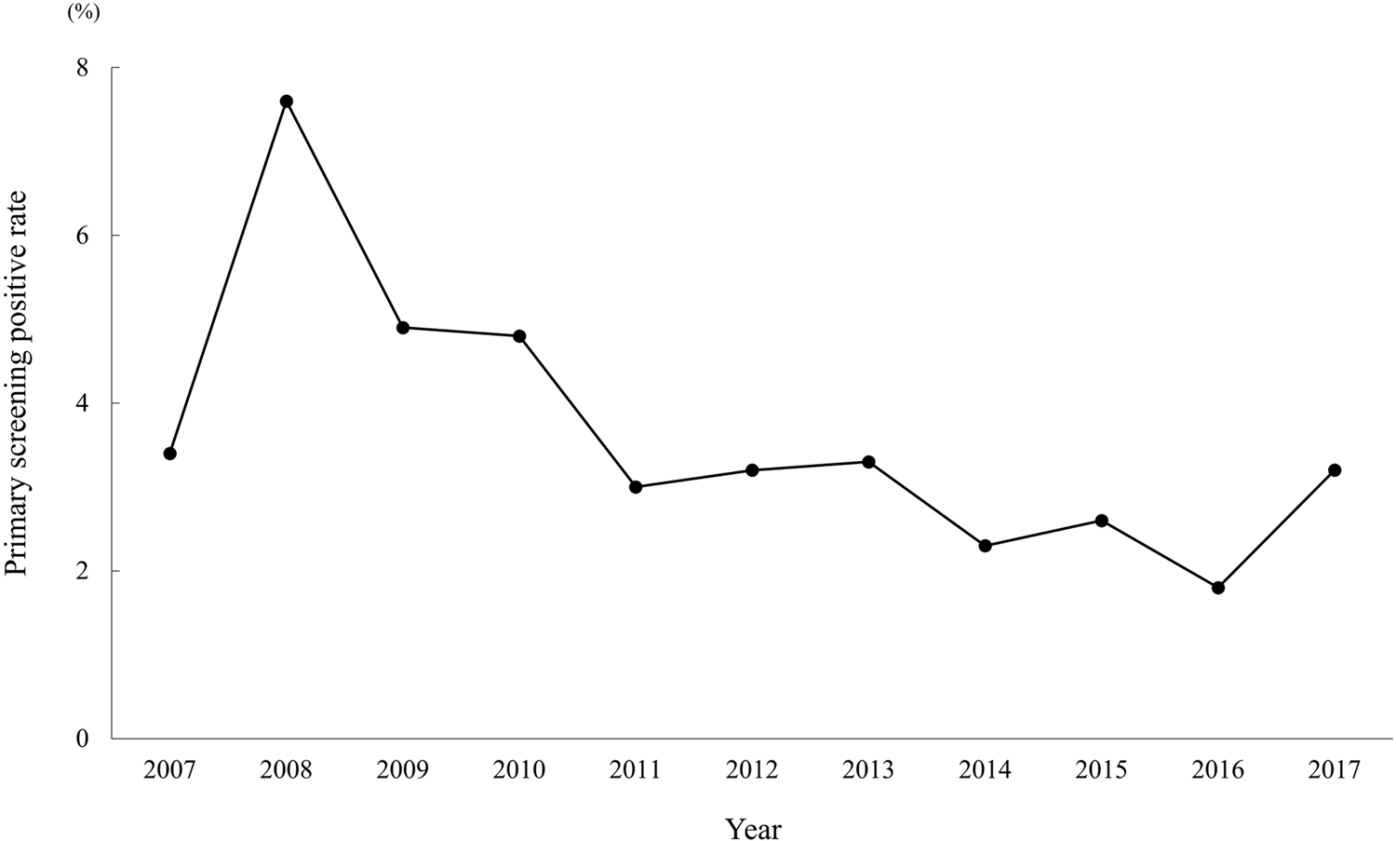
Prevalence trend of primary screening-positive rate

Between 2007 and 2017, 5178 (2343 boys and 2835 girls) of 5193 students (2349 boys and 2844 girls) (99.7%) underwent primary screening by a urinary antibody test (Table 1). The primary screening participation rate increased significantly from 99.5% in the early period to 99.9% in the late period (*p* = 0.02). Fourteen students (3.4%) were positive in primary screening in 2007, and the positive rate remained at 1.8–7.6% thereafter. The overall prevalence of *H.* *pylori* antibody positivity was 3.6% (184 students; 88 boys and 96 girls) during the 11 years (Fig. 1). Compared with the positive rate of 4.7% in the early period, the 2.8% in the late period was significantly lower (*p* < 0.01). One hundred-three (56%; 45 boys and 58 girls) of the 184 students who were positive in primary screening visited our hospital for secondary screening (Table 2). All 103 students underwent EGD with histological evaluation and culture analysis, and 90 (87.4%; 43 boys and 47 girls) were diagnosed as having *H. pylori* infection. The secondary test positive rate increased significantly from 78% in the early period to 96.2% in the late period (*p* = 0.01).

**Table 1:** Primary screening participation rate and positive rate

Year	Primary screening participation rate n, (%)			Primary screening-positive rate n, (%)		
	Each year	Early period/Late period	*p*-value	Each year	Early period / Late period	*p*-value
2007	409/414 (98.8)	2102/2113 (99.5)	0.02	14/409 (3.4)	99/2102 (4.7)	< 0.01
2008	370/373 (99.2)			28/370 (7.6)		
2009	445/445 (100)			22/445 (4.9)		
2010	478/480 (99.6)			23/478 (4.8)		
2011	400/401 (99.8)			12/400 (3.0)		
2012	539/539 (100)	3076/3080 (99.9)		17/539 (3.2)	85/3076 (2.8)	
2013	610/611 (99.8)			20/610 (3.3)		
2014	516/518 (99.6)			12/516 (2.3)		
2015	530/531 (99.8)			14/530 (2.6)		
2016	449/449 (100)			8/449 (1.8)		
2017	432/432 (100)			14/432 (3.2)		
Total	5178/5193 (99.7)	–	–	184/5178 (3.6)	–	–

**Table 2:** Secondary screening participation rate and positive rate

Year	Secondary screening participation rate *n*, (%)			Secondary screening-positive rate n, (%)		
	Each year	Early period/Late period	*p*-value	Each year	Early period/Late period	*p*-value
2007	9/14 (64.3)	50/99 (50.5)	0.11	9/9 (100)	39/50 (78)	0.01
2008	14/28 (50)			10/14 (71.4)		
2009	7/22 (31.8)			5/7 (71.4)		
2010	11/23 (47.8)			7/11 (63.6)		
2011	9/12 (75)			8/9 (88.9)		
2012	10/17 (58.8)	53/85 (62.4)		10/10 (100)	51/53 (96.2)	
2013	12/20 (60)			11/12 (91.7)		
2014	9/12 (75)			8/9 (88.9)		
2015	10/14 (71.4)			10/10 (100)		
2016	4/8 (50)			4/4 (100)		
2017	8/14 (57.1)			8/8 (100)		
Total	103/184 (56)	–	–	90/103 (87.4)	–	–

### Endoscopic findings

The most frequent endoscopic finding was nodular gastritis in 75 of 90 infected students (83.3%) (Table 3). Atrophic gastritis was observed in 60 students (66.7%). All cases of atrophic gastritis were closed type (C-1: 14, C-2: 37, C-3: 9) according to Kimura–Takemoto classification, with no open type cases.

**Table 3:** Endoscopic findings in students with *Helicobacter pylori* infection (*n* = 90)

	n, (%)
Nodular gastritis	75 (83.3)
Atrophic gastritis C-1: C-2: C-3	60 (66.7) 14: 37: 9
Duodenal ulcer scar	6 (6.7)
Duodenal erosion	4 (4.4)
Gastric ulcer scar	1 (1.1)

Atrophy was judged according to Kimura–Takemoto classification

Histological findings.

**Fig. 2:**
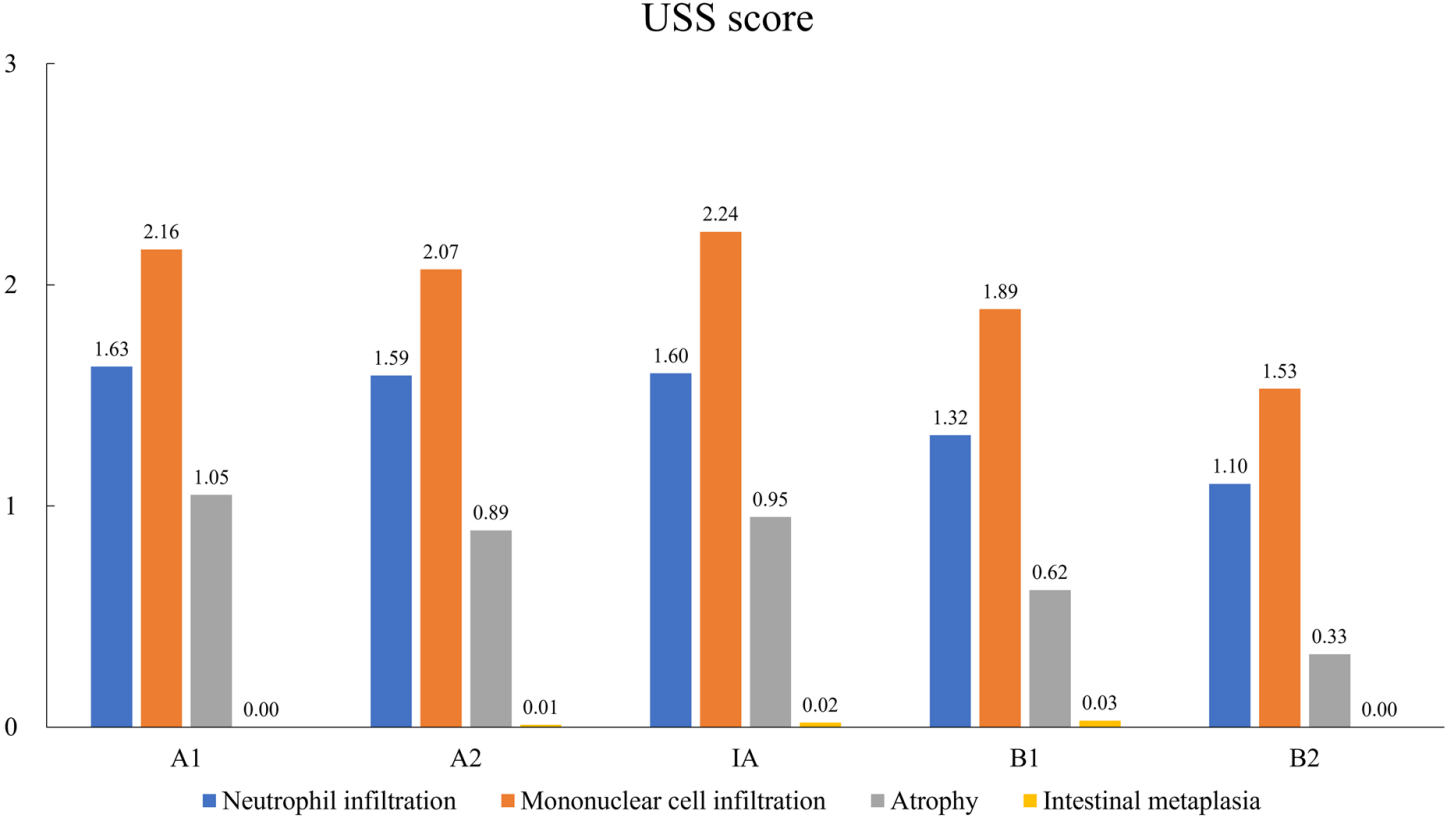
Histological updated Sydney System (USS) scores. Data are presented as the mean

The mean USS scores for neutrophilic and mononuclear cell infiltration at each site ranged from 1.10 to 1.63 and 1.53 to 2.24, respectively (Fig. 2). The mean USS score for atrophy was 0.33–1.05, indicating mild atrophy. Several cases also displayed intestinal metaplasia.

Susceptibility of *H. pylori* to CAM and MNZ.

CAM and MNZ resistance were identified in 37 (41.1%) and 41 (45.6%) of 90 students, respectively (Table 4). We observed no difference in those resistance rates between the early and late periods (*p* = 0.38 and *p* = 0.45, respectively).

**Table 4:** Susceptibility of *Helicobacter pylori* to antibiotics

Year	Clarithromycin resistance rate *n*, (%)			Metronidazole resistance rate *n*, (%)		
	Each year	Early period/Late period	*p*-value	Each year	Early period/Late period	*p*-value
2007	1/9 (11.1)	14/39 (35.9)	0.38	3/9 (33.3)	16/39 (41)	0.45
2008	2/10 (20)			2/10 (20)		
2009	3/5 (60)			5/5 (100)		
2010	4/7 (57.1)			4/7 (57.1)		
2011	4/8 (50)			2/8 (25)		
2012	5/10 (50)	23/51 (45.1)		4/10 (40)	25/51 (49)	
2013	5/11 (45.5)			3/11 (27.3)		
2014	4/8 (50)			4/8 (50)		
2015	4/10 (40)			7/10 (70)		
2016	1/4 (25)			1/4 (25)		
2017	4/8 (50)			6/8 (75)		
Total	37/90 (41.1)	–	–	41/90 (45.6)	–	–

### Eradication therapy outcomes

All 90 students with *H. pylori* infection received eradication therapy, with an overall initial eradication success rate of 96.5% (82 students) among the 85 students who completed treatment evaluation (Table 5). The remaining 5 students did not undergo post-therapy evaluation and were considered lost to follow-up. The success rates against *H. pylori* by eradication regimen were 94% (47 of 50) for CAM and 100% (35 of 35) for MNZ, with no significant difference between the regimens (*p* = 0.38). The eradication success rates using PPI- or P-CAB-based regimens were 95.9% (71 of 74) for PPI and 100% (11 of 11) for P-CAB, which were also statistically comparable (*p* = 0.84). The 3 students with unsuccessful initial eradication therapy achieved eradication by secondary treatment using the MNZ-based regimen. Adverse events associated with eradication therapy were observed in 11 of 85 students (12.9%), consisting of skin rash in 5 students, soft stool/diarrhea in 3 students, and nausea/vomiting in 3 students. One student with a skin rash required hospitalization. No student discontinued eradication therapy due to adverse events.

**Table 5:** Eradication therapy outcomes (*n* = 85)

	*n*, (%)	*p*-value
Initial eradication therapy success rate	82/85 (96.5)	
CAM-based regimen MNZ-based regimen	47/50 (94)^*^ 35/35 (100)	0.38
PPI-based regimen P-CAB-based regimen	71/74 (95.9) 11/11 (100)	0.84
Adverse events Skin rash Soft stool/diarrhea Nausea/vomiting	11/85 (12.9) 5 (5.9) 3 (3.5) 3 (3.5)	–

CAM, clarithromycin; MNZ, metronidazole; PPI, proton pump inhibitor; P-CAB, potassium-competitive acid blocker

^*^3 students with unsuccessful initial eradication therapy using a clarithromycin-based regimen achieved eradication by secondary treatment using a metronidazole-based regimen

## Discussion

*H. pylori* eradication therapy has been established to prevent gastric cancer and other *H. pylori*-related diseases [15]. However, it also remains necessary to identify *H. pylori*-infected young people despite low infection rates and confirm the usefulness and safety of eradication therapy. To address this issue, we trialled *H. pylori* screening using a urinary anti-*H. pylori* antibody test in school health checkups for 11 years. Our findings revealed very high compliance to initial testing, several unique clinicopathological characteristics, and the effectiveness of *H. pylori* resistance-based treatment.

The primary screening participation rate was extremely high (99.7%) for the entire study period. Urine testing is common in school health screenings, and the use of a urine sample is considered a non-invasive way to promote participation. Our participation rate was higher than the 61.7–97.3% in previous studies of teenagers [6–8, 16], which cited a lack of interest for refusing to participate [16]. Carefully educating high school students and their parents about *H. pylori* infection may help heighten their understanding and willingness to consent. Indeed, the significant increase in participation rate in the late period suggested greater comprehension through the yearly continuation of *H. pylori* screening. The urinary anti-*H. pylori* antibody-positive rate over the 11-year period mostly remained below 5%, with the *H. pylori* infection rate low as in previous reports on adolescents [6–8, 16]. The infection rate among children was presumed to decrease annually [17], which was evident by the significant decline in the late period. Further decreases may be expected over time. We observed that the secondary test positive rate was significantly higher in the late period than in the early period. The immunochromatography method employed for measuring urinary anti-*H. pylori* antibodies as the primary screening in this study can be influenced by visual interpretation and the presence of proteinuria [10, 18]. It is possible that these factors affected the early primary screening results more than the later ones although the exact reason for the difference in rates is uncertain. Taken together, the introduction of *H. pylori* screening into school health checkups may be effective in identifying infected youths owing to a very high participation rate.

On the other hand, only 56% of primary screening-positive students underwent secondary screening following recommendations by a school doctor, with the remaining 44% lost to follow-up. Endoscopy was employed as additional screening at our hospital, which might have been perceived as overly invasive for the students. Thus, the time and the effort required for secondary screening along with the invasiveness of endoscopic examinations may have influenced our results. In another report, *H. pylori* status was judged by a urea breath test as secondary screening by the so called “test-and-treat strategy” [8], and so whether endoscopy is unnecessary remains to be determined. Future considerations to increase follow-through to secondary screening may include a hybrid strategy, with the option of either EGD or a urea breath test after careful explanation to the student and parents.

Although the purpose of endoscopy in this study was to more precisely investigate the actual status of upper gastrointestinal lesions and *H. pylori* susceptibility trends in young students, we still advocate EGD as secondary screening in clinical practice for several reasons. First, we were able to perform culture tests using the obtained biopsy samples, which improved eradication success rates. Our study showed a high CAM resistance rate of 41% during the 11-year period. CAM resistance of *H. pylori* among children in Japan has been increasing, with published rates of 40.7% from 2003 to 2007 [19], 52.6% from 2007 to 2012, and 84.6% from 2013 to 2018 [20]. In our cohort, CAM resistance rate increased from 35.9% in the early period to 45.1% in the late period. Despite being lower than figures reported in the last decade, CAM resistance among young people was consistently high, with some years over 50%. The MNZ resistance rate in this investigation was also high at 45.6% and comparable to a report of 53% among children in East Asia from 2005 to 2015 [21]. Globally, it has been established that eradication regimens should achieve ≥ 90% efficacy [22]. Although the CAM resistance rate of *H. pylori* is currently considered the main cause of eradication treatment failure [23], we could achieve a high initial eradication success rate of 96.5% by selecting the initial regimen based on the results of susceptibility testing. CAM has virtually no antibacterial effect against CAM-resistant strains, whereas MNZ continues to exhibit some effects against MNZ-resistant strains [24]. Therefore, a method of eradication therapy exclusively with a MNZ-based regimen without susceptibility testing is possible. However, MNZ is reportedly carcinogenic in animals and mutagenic in vitro [25]. While unlikely to increase the risk of carcinogenesis in humans, MNZ should be used conservatively due to the lack of long-term data [26]. Recently, P-CAB-based triple therapy was found to be more effective than PPI-based regimens for first-line *H. pylori* eradication therapy, even against CAM-resistant strains [27]. However, the success rate of P-CAB-based first-line eradication in junior high school students was less than 90% at 83.8–85.7% [6, 7, 28–30]. In the present study, we observed no significant therapeutic differences between PPI-based and P-CAB-based regimens, with both achieving high eradication success rates. Therefore, it still appears desirable to select the *H. pylori* eradication regimen after testing for CAM resistance rather a default PPI-based or P-CAB-based approach.

Another merit of endoscopic approaches to secondary screening is that clinicians can diagnose gastrointestinal diseases and evaluate the degree of inflammation of the gastric body, which is considered a carcinogenic risk factor for diffuse-type gastric cancer in nodular gastritis [1, 31, 32], as well as the extent of atrophy and intestinal metaplasia as risk factors for differentiated gastric cancer [33] using biopsy samples. In this study, the most common endoscopic finding in *H. pylori*-infected students was nodular gastritis (83.3%), with several cases of gastroduodenal ulcer scars. Although rare, gastric cancer associated with *H. pylori* has been reported in children and adolescents [34]. Since young people are generally at low risk of carcinogenesis, however, the follow-up protocol after eradication has not yet been established. We earlier described a case of advanced gastric cancer detected 3 years after eradication for nodular gastritis in a student subject [35], which highlighted that gastric cancer after eradication was rare, but possible, in young patients. We have also reported that young patients with nodular gastritis may have a low risk of carcinogenesis due to fewer inflammatory changes in the gastric body versus afflicted adult patients [36]; however, the severe inflammation detected in some youths suggested a future possibility of cancer [36]. Indeed, the above case of advanced gastric cancer 3 years after eradication had shown moderate inflammation in the gastric body in histological evaluations before eradication therapy [35]. A small number of cases already showed histological atrophy of the gastric body and intestinal metaplasia in this study. International consensus advocates that the *H. pylori* test-and-treat program should ideally target a younger adult population, such as individuals 20–40 years of age, before any potential preneoplastic changes in the gastric mucosa [22]. Considering the histological results of this study, intervention also appears beneficial in teenagers. Although risk stratification for gastric cancer after eradication based on pre-treatment histological analysis has been proposed [37, 38], there are as yet no reports on young patients or follow-up protocol. Endoscopic biopsy sampling of the gastric mucosa to assess histological findings before eradication may help predict future carcinogenesis and establish a more optimal follow-up strategy. However, if *H. pylori* school screening is to be implemented nationwide with EGD as secondary screening, several practical considerations must first be resolved, such as cost, capacity of endoscopy, and perceived invasiveness. Future multi-center prospective studies are warranted.

Adverse events associated with eradication therapy were observed in 12.9% of students. Skin rash was the most common event, while soft stool/diarrhea, another known side effect, was the second most common event. Adverse events in children and adolescents treated with PPI- or P-CAB-based triple therapy range widely from 4.0% to 66.2%, with soft stool/diarrhea ranging from 1.4% to 36%, and skin rash ranging from 0.1% to 4.5% [6, 7, 23, 28–30, 39, 40]. The incidences of adverse events observed in this study were comparably frequent but ranked differently. However, since the survey was conducted by interview only, there was a risk of underreporting if a student did not mention any events. In our intergenerational comparison of eradication therapy, younger patients exhibited more frequent and severe adverse events compared with middle-aged and older patients [41]. As a student required hospitalization in the present cohort, special attention to skin rash and other adverse events is advised during eradication therapy in young patients. Susceptibility testing to increase the initial eradication rate will also help complete treatment in a single session and reduce the risk of complications, which has been recommended in pediatric guidelines [42].

There were several limitations to this study. First, as it was conducted at a single high school, a larger scale survey is needed to fully consider the introduction of this trial into the nationwide school screening system. Second, no follow-up of students after *H. pylori* eradication was conducted; long-term monitoring is necessary to clarify the effect of eradication in young people on gastric cancer prevention. Nonetheless, we believe that this study remains meaningful since it sheds light on the trends in *H. pylori* infection rates, the clinicopathological characteristics of *H. pylori*-infected students including endoscopic findings, and the usefulness and the safety of eradication therapy over an 11-year period.

In conclusion, the introduction of primary screening in high school health checkups helped clarify the clinicopathological characteristics of *H. pylori*-infected students and the results of eradication therapy. Although it will be necessary to improve the secondary screening participation rate and contend with antibiotic resistance, this project exemplifies the utility of introducing *H. pylori* screening and eradication in youths, which may help prevent gastric cancer.
